# Exploring Evaluation Variables for Low-Cost Particulate Matter Monitors to Assess Occupational Exposure

**DOI:** 10.3390/ijerph17228602

**Published:** 2020-11-19

**Authors:** Sander Ruiter, Eelco Kuijpers, John Saunders, John Snawder, Nick Warren, Jean-Philippe Gorce, Marcus Blom, Tanja Krone, Delphine Bard, Anjoeka Pronk, Emanuele Cauda

**Affiliations:** 1 Netherlands Organization for Applied Scientific Research (TNO), 3584 CB Utrecht, The Netherlands; Eelco.kuijpers@tno.nl (E.K.); Marcus.blom@tno.nl (M.B.); tanja.krone@tno.nl (T.K.); anjoeka.pronk@tno.nl (A.P.); 2 Health and Safety Executive (HSE), HSE Science and Research Centre, Harpur Hill, Buxton SK17 9JN, UK; John.Saunders@hse.gov.uk (J.S.); Nick.Warren@hse.gov.uk (N.W.); Jean-Philippe.Gorce@hse.gov.uk (J.-P.G.); Delphine.Bard@hse.gov.uk (D.B.); 3 Centers for Disease Control and Prevention, National Institute for Occupational Safety and Health (NIOSH), 1090 Tusculum Avenue, Cincinnati, OH 45226, USA; jts5@cdc.gov (J.S.); cuu5@cdc.gov (E.C.)

**Keywords:** low-cost monitors, wearables, sensors, evaluation, occupational, exposure monitoring, particulate matter

## Abstract

(1) Background: Small, lightweight, low-cost optical particulate matter (PM) monitors are becoming popular in the field of occupational exposure monitoring, because these devices allow for real-time static measurements to be collected at multiple locations throughout a work site as well as being used as wearables providing personal exposure estimates. Prior to deployment, devices should be evaluated to optimize and quantify measurement accuracy. However, this can turn out to be difficult, as no standardized methods are yet available and different deployments may require different evaluation procedures. To gain insight in the relevance of different variables that may affect the monitor readings, six PM monitors were selected based on current availability and evaluated in the laboratory; (2) Methods: Existing strategies that were judged appropriate for the evaluation of PM monitors were reviewed and seven evaluation variables were selected, namely the type of dust, within- and between-device variations, nature of the power supply, temperature, relative humidity, and exposure pattern (peak and constant). Each variable was tested and analyzed individually and, if found to affect the readings significantly, included in a final correction model specific to each monitor. Finally, the accuracy for each monitor after correction was calculated; (3) Results: The reference materials and exposure patterns were found to be main factors needing correction for most monitors. One PM monitor was found to be sufficiently accurate at concentrations up to 2000 µg/m^3^ PM_2.5_, with other monitors appropriate at lower concentrations. The average accuracy increased by up to three-fold compared to when the correction model did not include evaluation variables; (4) Conclusions: Laboratory evaluation and readings correction can greatly increase the accuracy of PM monitors and set boundaries for appropriate use. However, this requires identifying the relevant evaluation variables, which are heavily reliant on how the monitors are used in the workplace. This, together with the lack of current consensus on standardized procedures, shows the need for harmonized PM monitor evaluation methods for occupational exposure monitoring.

## 1. Introduction

Low-cost lightweight optical monitors for measuring airborne particulate matter (PM) concentrations in near real-time are becoming available and may be applied to a multitude of purposes, including occupational exposure monitoring [1,2]. The term “monitor” is used here to refer to either the sensing part of any monitor (e.g., optical sensing elements of an optical particle counter and photometer) or the monitor itself (complete, ready-to-use device that generally includes a battery, data storage or communication system and one or more sensors). Low-cost optical PM monitors (hereafter referred to as simply “PM monitors”) could potentially provide substantial benefits to the monitoring of PM in occupational settings compared to traditional gravimetric PM exposure assessment methods, which rely on sampling pumps and filters. These methods are based on collecting PM on a filter followed by gravimetrical analysis. One advantage of these PM monitors is the ability to collect data at greater spatiotemporal resolutions (measurements every few seconds compared to one average concentration over several hours). This higher time-resolved data may provide new insights into exposure dynamics. For instance, knowing whether exposure occurs during specific events or is constant throughout the day can be used to deploy control measures more effectively [3]. Also, most monitors can report and process the data as soon as it is collected and while the instrument is still deployed. This allows direct feedback and intervention systems that can immediately prevent further exposure. Once deployed, this technology requires minimal efforts at lower cost compared to traditional sampling methods which have additional analytical and labor costs. In addition, the acquisition costs are considerably lower compared to currently used real-time devices in occupational settings (roughly thousands of euros for validated devices such as photometers and up to tens of thousands for validated research-grade equipment), which allows more units to be deployed simultaneously for comprehensive spatial exposure monitoring. A more detailed description of the potential applications of low-cost monitors is given by Goede et al. (2020, manuscript in press). The reduced cost, ease of deployment, direct reading capabilities together with the wireless network ability of this technology mean that new ways of collecting and sharing occupational hygiene information between employers, employees and regulators become possible.

Despite the new opportunities and potential advantages, the use of low-cost monitors for exposure monitoring also brings new challenges [2]. Generally, PM monitors are less accurate compared to high-end devices [4,5], the reported size fractions are generally different from workplace occupational relevant fractions and once deployed it becomes difficult to confirm remotely that monitors remain in calibration, in working order, and have not been tampered with. Therefore, we did not expect the current technology to be suitable for exposure assessment studies nor for direct comparison with occupational exposure limits, but nonetheless to be particularly useful for detecting exposure events in the workplace as they occur. Although accurate measurements are important for monitoring occupational exposure, the previously described advantages may outweigh some of these disadvantages. To gain insight in the accuracy of the PM monitors, an evaluation should be performed [4]. During the evaluation, the PM monitor readings may be corrected to better align with a trusted reference. However, this basal correction may not be sufficient if internal and external factors are at play that could affect the monitor readings. The impact of these factors on the monitor readings may be corrected for using a statistical model, which will be defined thereafter as a correction model.

The accuracy of PM monitors has been investigated in several scientific studies, mainly in environmental settings [6,7,8,9,10] with a few in occupational settings [1,11]. However, no evaluation methods have been described for PM monitors to be deployed in the workplace. This is in part because the evaluation processes must be developed and optimized for each target workplace process taking place. This is required because monitor responses are likely to differ for different substances (e.g., due to varying particle size distributions and refractive indices), concentration ranges, exposure dynamics, and environmental conditions [11,12,13]. The large variability in the conditions and processes found in the workplace are the main challenge faced compared to environmental purposes. Because of the unique characteristics of the workplace, environmental evaluation protocols are only partly suitable for occupational settings.

The aim of this study was to perform an evaluation of a selection of PM monitors to be used for monitoring occupational exposure situations. More specifically, this involved identifying from literature evaluation variables (e.g., environmental variables such as temperature and relative humidity) related to possible workplace conditions and likely to affect monitors readings, testing them experimentally and including them in correction models. To determine the performance of each correction model, and therefore each PM monitor output after correction, the final accuracy was calculated. The added value of the evaluation was further investigated by comparing the accuracies of each correction model with and without the evaluation variables, and therefore demonstrating to which extent correcting, for example, for the effect of temperature and relative humidity on the monitors readings improve the monitors output. This study is a first step towards the development of an appropriate and fit-for-purpose evaluation strategy for the successful deployment of low-cost PM optical monitors in the workplace.

## 2. Methods

The evaluation was based on the methods described by Spinelle et al. [14], NIOSH [15], CEN Standards 16013–1:3 [16,17,18], and ISO Standard 11095 [19]. From these sources of information, “evaluation variables” was defined as a term that describes the environmental or non-environmental conditions that can vary in the workplace and may affect the monitor readings. Examples are the measured material, drift, and humidity. The evaluation described in this study focuses on the effects of different evaluation variables on the accuracy of the monitors.

To gain insight in the different evaluation needs for different types of devices, six distinct PM monitors were tested. Experiments were conducted in a customized exposure chamber where the evaluation variables could be controlled and adjusted to relevant levels. Results from traditional gravimetric analysis after sampling of the airborne particles and readings from laboratory grade real-time equipment were used as reference measurements.

Monitors were simultaneously investigated in the chamber. Data preparation steps included defining concentration limits for the PM monitors (e.g., at which concentration the output was not reliable) and normalization of the dataset, which was required for the subsequent analysis. Modelling approaches were used to assess each evaluation variable individually, as well as for the generation of the final correction models. These models use the PM monitor measurements, correct for the evaluation variables and predict the “true” reference concentration determined by an aerodynamic particle sizer (APS from TSI, Shoreview, MN, USA). The final accuracy of the monitors was calculated considering the bias (the deviation of the measurements from the “true” concentration) and precision (random spread of measurements) using a dataset in which the levels of multiple evaluation variables varied.

### 2.1. Experimental Design

A typical evaluation should be undertaken for a specific workplace and/or task and requires the experimental situation to mimic the target field condition as closely as possible. Since this study is focusing on a hypothetical workplace situation, the experimental design was based on considerations that were generally required for use in the workplace according to the authors. This included the concentration range, target accuracy and target responsiveness, as well as the evaluation variables (parameters such as temperature and humidity that may affect the monitor readings). The experimental design is summarized in Table 1 and described below.

#### 2.1.1. Concentration Range

The PM concentration range to which the monitors are to be exposed in the laboratory evaluation should include the range for which the workplace measurements would be expected to be accurate (or rather, above which value quantification is no longer relevant). The monitors are likely to be more accurate if readings are confined within smaller concentration ranges.

Experiments were performed with PM concentrations up to 5000 µg/m^3^ of respirable fraction, equivalent to the Dutch occupational exposure limit for unspecified respirable dust [20].

#### 2.1.2. Target Accuracy

The target accuracy is the set value representing how accurate each monitor needs to be for an application. The target accuracy for the PM monitors evaluated here was set at ≤50% of the reference value, which would be expected to be sufficient for indicative measurements. This value is derived from the EU Air Quality Directive 2008/50/EC [21]. The final accuracy was calculated experimentally after applying the correction model using a dataset with variations in evaluation variables.

#### 2.1.3. Target Responsiveness

The target responsiveness is the time that a monitor takes to span from the minimum PM concentration of the concentration range to 80% of the maximum, or vice versa [14]. This is relevant if rapid changes in PM concentrations are expected in the workplace and need to be measured. The target responsiveness was arbitrarily set at one minute.

Evaluation variables are parameters that should be integrated into the correction model, if demonstrated to have a significant effect on the measurements. Evaluation variables are summarized in Table 2 and described below.

#### 2.1.4. Reference Material

During the laboratory experiments, the monitors were exposed to PM generated from reference materials dispersed in a controlled test chamber. PM monitors may react differently to the different PM generated, and the reference materials used can have a large effect on the PM monitor readings and accuracy. The reference material(s) should be selected carefully to be as similar as possible to the aerosols that are expected in the workplace [19]. By doing so, the laboratory correction model is more likely to provide the best fit for the readings once the monitors are deployed in the workplace. In addition, the efforts required for the development of the field correction model can be contained and reduced.

In this study, instead of selecting a single material characteristic of the target workplace, three reference materials were selected and tested: Arizona Road Dust, Quartz (>97% SiO_2_) and Al_2_O_3_ (all KSL Staubtechnik GmbH, Lauingen, Germany). These three reference materials allowed testing for different chemical and optical properties (e.g., density and refractive index), but not particle size distribution, since all materials conformed to the A2 ISO 12103–1 size distribution. Results were used to give an indication of the effects of the materials characteristics on the PM monitors readings. To this end, experiments were performed with each reference material.

#### 2.1.5. Within-Device Variation (Short-Term Drift)

The responses of the PM monitors may decrease over repeated exposures, for instance because the optical elements became contaminated over time, which could lead to erroneous measurements [14]. Therefore, within-device temporal variation was tested as the systematic differences between repeated experiments. Experiments for this variable were performed at time 0 h and repeated after ~24 and ~48 h.

It should be noted that long-term drift (or recalibration interval) should also be tested. However, since this is best done periodically while the monitors are deployed in the field, this is not included in the current study.

#### 2.1.6. Between-Device Variation

Different physical units of the same PM monitor type may respond differently to the same concentrations of PM due to random variations in the production process. Therefore, multiple PM monitors should be tested in parallel and possible device-specific effects adjusted for in the correction model [19]. Between-device variability was tested using at least three units of each PM monitor type during all experiments.

#### 2.1.7. Power Supply

The different options for power supply may have an effect on the monitor readings [15]. Therefore, this was tested by repeating exposures while PM monitors were battery powered and while wired powered.

#### 2.1.8. Temperature

Temperature may have an effect on the sensitivity of the sensing elements in the PM monitor and was thus included as an evaluation variable [17]. Three temperatures were tested and included 15, 20, and 25 °C thought to be representative of an indoor workplace environment.

#### 2.1.9. Relative Humidity

The environmental humidity can affect the PM monitor indirectly by changing properties such as the reflectivity and shape of particulates [22]. This can lead to erroneous measurements. The levels of relative humidity tested were 25, 50, and 75%, depictive of an indoor environment.

#### 2.1.10. Exposure Pattern

During the experiments, the PM monitors were exposed to the aerosol. Exposure in the chamber could be kept at one constant concentration for the whole experiment (stable) or the concentration could fluctuate from the concentration-minimum to the -maximum and back to the -minimum, mimicking exposure “peaks” (transient). Both can occur in the workplace and monitors may react differently to these exposure patterns.

To test this evaluation variable, two exposure patterns were tested at the concentration range defined earlier (e.g., 0–5000 µm/m^3^ respirable fraction).

The constant concentration (plateau) was kept for ≥15 min (often longer due to sample collection for gravimetric analysis) at approximately 333, 666, 1000, 2500, and 5000 µg/m^3^.

The transient exposures consisted of three “peaks”, a peak being defined by a concentration change from 0 to approximately 5000 and back to 0 µg/m^3^ in about 30 min.

### 2.2. Experimental Setup

The experimental setup used to undertake the experiments is schematically depicted in Figure S1. The size of the exposure chamber is about 80 × 80 × 80 cm^3^ and is capable of controlling temperature between 15 and 25 °C and humidity between 25 and 75%. The validity of the setup was confirmed experimentally by testing the correlation between measurements collected using an APS and results of gravimetrical sampling (see details of sampler in *Reference measurements*) for different reference materials and concentrations. PM homogeneity in the exposure chamber was investigated by comparing the APS measurements taken at multiple positions.

The seven evaluation variables were tested individually. This was done by repeatedly exposing monitors to PM in the controlled environment, where only one variable was changed (e.g., three repeated experiments at low, medium and high temperature). A total of 43 experiments were conducted (see Table S1).

### 2.3. Low-Cost Optical PM Monitor Selection

Six PM monitors (3 units each) were tested. Their technical specifications are reported in Table 3. Selection was based on consumer readiness, capacities to continuously measure for a prolonged period of time (such as an 8 h shift) and at high sampling time frequency (more than one reading per minute) and practical consideration that make them suitable for use in the workplace (user-friendly, size, weight, etc.). No low-cost monitors were available that reported respirable or inhalable size fractions as defined for occupational settings. Therefore, the PM monitors were selected on the capability to measure PM at a size of 2.5 µm and smaller (PM_2.5_), since this is closest to the respirable fraction that is used when considering potential human health effects in occupational settings [23].

### 2.4. Reference Measurements

Reference measurements were provided by duplicate gravimetrical samplers consisting of GilAir personal air sampling pump (Sensidyne, St. Petersburg, FL, USA) combined with SKC cyclone sampler 225–69 (SKC Ltd., Dorset, UK), sampling at 3 L/min. For the study, this was considered to be the “gold standard” measurement method for respirable dust that was used to correct the real-time reference APS time-average values and PM monitors concentrations outputs (once transformed into time-average values). A total of 36 gravimetric samples were collected. The APS was used as a real-time reference device. APS PM_2.5_ concentrations were corrected for material density and particle dynamic shape factor per reference material, as recommended by the manufacturer. In short, the particle mass was calculated using the mean particle size bin of the APS as particle size and density of the material. Particle mass was corrected using the dynamic shape factor and the total mass was calculated using the number of counts by the APS. The shape factor for all materials was 1.5 and the densities were 2.6, 2.6, and 3.95 g/cm^3^ for ARD, SiO_2_ and Al_2_O_3_, respectively. Sampling time was set at 15 s. For comparison with gravimetrical samples, APS measurements were corrected based on the respirable convention according to the ISO 7708 standard [24]. All reference measurements are performed in the middle of the exposure chamber.

### 2.5. Statistical Analysis

All analyses were performed using R version 3.6.1 [25].

#### 2.5.1. Confirmation of Experimental Setup and APS Reference

Validity of the APS reference and experimental setup was performed by developing a linear statistical correction model based on APS average values and gravimetrical sample results at default environmental settings (20 °C, 50% RH) for transient and stable exposures. This model was used to correct structural differences between the correction methods. A similar model was generated for experiments during which the temperature, humidity and exposure pattern were varied. This was used to confirm that changes in these variables had no effect on the APS measurements. The correlation of both models was used to confirm that the APS measurements were reliable at all concentrations.

#### 2.5.2. PM Monitor Data Preparation

Negative correlations between the PM monitor and the reference were identified and removed by fitting a local regression on each experimental data set using the loess function in base R. Where the curve followed a half-circle shape (e.g., one x-value related to two y-values), a negative correlation was present. We wanted to investigate only structural miscorrelations, and not outliers, therefore only 10% of the experiments showing a negative correlation were selected for analysis. For these monitors, the highest readings for which no negative correlations were present were pooled. From this, the 10th percentile was used as the maximal reliable concentration that the PM monitor could report. The cutoffs for this analysis were set arbitrarily to form a conservative balance between correctness and data preservation.

For linear regression and linear mixed-effect modelling, linear responses between PM monitors and the APS reference are required. To this end, the PM monitor and APS measurement values were normalized. Testing of the most optimal normalization method was performed using the bestNormalize function from the R-package with the same name [26].

#### 2.5.3. Variable Testing and Correction Model Development

Linear mixed-effect models were used for the correction model since some of the evaluation variables were random (e.g., values depend on a random phenomenon) and some are fixed (e.g., values are structured and can be predicted on forehand). These models were generated using the lme4 package [27]. When no random effects were included, linear regression models were developed using the functions in base R.

Evaluation variables were first tested separately, with all other variables at average values (sensors operated on battery, 20 °C, 50% RH and transient exposure pattern with peaks at maximum concentration of about 5000 μg/cm^3^). On this data subset, two models were fitted; one including the tested variable and one without. The effect of the variable was assessed by comparing the two models using three criteria. First, an analysis of variance (ANOVA) between the models was performed to test that the variable has a significant contribution to the correction model (*p* < 0.05). Second, the quality of the model had to be improved, which was shown as a decrease of the Akaike’s information criterion (AIC). This criterion balances model sparsity and goodness of fit, which prevents the need for collecting variables in the workplace that only slightly increase the model accuracy. Finally, the mean absolute error (MAE) of the models was calculated to test that the variable results in a more accurate prediction, where a smaller MAE shows a more accurate model. This measure was used to quantify the increase or decrease in PM monitor accuracy and therefore the importance of the evaluation variable. Variables which passed all three criteria were added into the final correction model, which was fitted on the experiments of all evaluation variables.

Some prioritization was required because not every combination of variables could be tested experimentally. Between-device variation was investigated first and, if an effect was found, corrected for in further analysis of the remaining independent variables by adding it to the mixed-effect models. The same was done for within-device variation and the reference materials. Between-device variation was tested as a random effect since the tested monitors were considered a random sample of a larger population of monitors, which would generally be the case in a field application (evaluation of a subset of monitors to be representative for all monitors used). Within-device time related variation (drift) was considered a fixed effect since this would be constant over time. The effect of each reference material was added to the models as a random effect, because the analyte in the target workplace can differ in chemical composition, refractive index, particle shape and size distribution. Temperature and relative humidity were tested as fixed effects, since these should have a constant effect on monitor performance. The exposure pattern was considered a fixed effect due to the two distinct usage options. Power supply was tested as a fixed variable since this should have constant effects on the monitor. Effects were included in the model as a slope, related to the PM monitor output.

#### 2.5.4. Correction Model Verification

Verification of the correction model was performed by calculating the uncertainty as described previously by NIOSH [15], which considers the uncertainty in the APS measurements (10%, as given by the manufacturer) as well as the instability in exposure concentrations. This method considers both the bias (average deviation of the PM monitor compared to the APS reference instrument) and precision (random spread in the monitor measurements) to calculate the accuracy, which is quantified as the relative uncertainty of the monitor measurements. Since this method requires bias and precision to be homogeneous, the verification dataset was divided into smaller concentration bins. Within these bins, the bias and precision are more homogeneous compared to the whole dataset. The verification was performed for each bin.

The verification dataset consisted of a full factorial design that included each possible combination of the tested materials, temperatures, and humidities. The verification was performed by calculating relative uncertainty measures of each low-cost monitor.

## 3. Results

Experimental procedures included the validation of the experimental setup, preparation of the data (data management steps), testing of the experimental parameters, development and verification of the correction model and assessment of the benefits of this procedure.

### 3.1. Validation of the Experimental Setup

**Figure 1:**
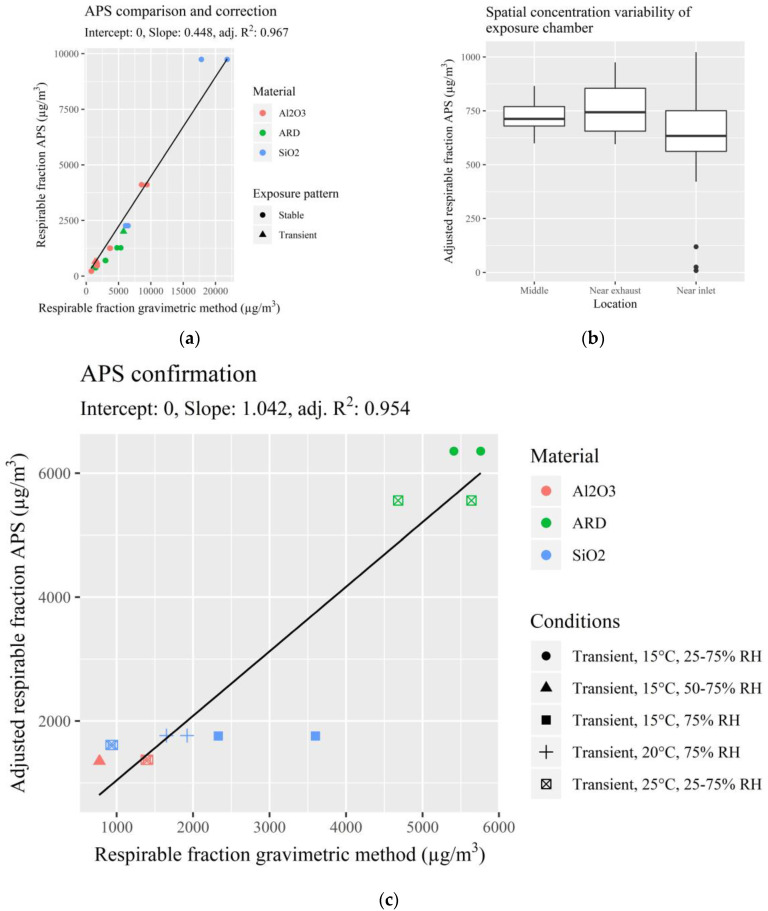
Preparative testing of equipment and test chamber environmental conditions. (**a**) Comparison and correction of an aerodynamic particle sizer (APS) respirable mass concentrations and respirable gravimetric concentrations; (**b**) testing of spatial variability within the exposure test chamber by comparing sampling locations using an APS; and (**c**) confirmation of adjusted APS respirable mass concentrations under changing evaluation variables.

Prior to the laboratory evaluation of the PM monitors, the experimental setup was validated and found to be appropriate for the evaluation step. Bias in the APS readings and repeatability of experiments were tested experimentally (Figure 1a). Linear regression showed that the APS underestimated the respirable PM concentrations compared to gravimetric samples. However, a high correlation (R^2^ = 0.97) confirmed that (after correction) the APS was consistent and accurate for the three materials tested and the two exposure patterns (stable and transient). During preliminary testing, concentrations above the testing concentrations (between 5000 and 20,000 μg/m^3^) were in line with the other concentrations so these were also included in the regression model. The spatial variability inside the chamber was also tested by changing the APS inlet location to ~10 cm after the inlet and ~10 cm before the exhaust during a constant concentration exposure period (Figure 1b). Median concentrations for the middle, near exhaust and near inlet were 713, 744, and 634 µg/m^3^, respectively. This shows that there were only slight differences in particle concentrations within the chamber. To reduce the effects of this, monitors were placed at different locations within the exposure chamber for each measurement day. Finally, transient exposure patterns were performed for each reference material with combinations of high, average or low temperature and relative humidity to investigate if these three parameters affected the corrected APS readings (Figure 1c). A linear regression showed that these experiments were comparable to the ones with temperature and relative humidity of 20 °C and 50% respectively (slope = 1.04, R^2^ = 0.95). One filter measurement showed a high concentration compared to a relatively low APS measurement, which is thought to be due to an artefact during the gravimetrical analysis.

### 3.2. Data Preparation

Visual inspection of the measurements revealed that in some cases a negative correlation occurred between the PM monitors and the reference APS instrument at PM_2.5_ concentrations. Therefore, for these instruments a maximum concentration without negative correlations were calculated and a cutoff was set for each device. Measurements above the cutoff were removed from the dataset.

**Figure 2:**
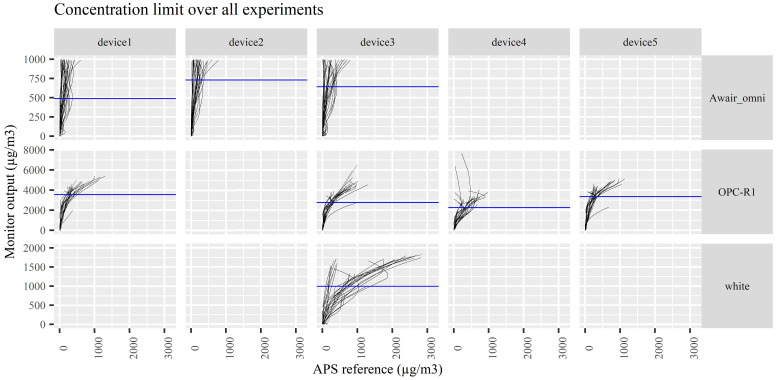
Concentration limit finding for the relevant units (negative correlation in ≥10% of experiments) of Awair omni, OPC-R1 and white prototype monitors. Correlation plots of the low-cost monitor PM_2.5_ compared to the APS reference PM_2.5_ fractions. Each experiment forms one line. Negative correlations show as a half-circle shape. The horizontal blue line depicts the calculated concentration limit, which is calculated as p10 of the individual concentration limits for each experiment.

All three Awair omni devices, four OPC-R1 devices and one “white” prototype device showed negative correlations with the reference instrument (Figure 2). For the Awair omni, the cutoffs were calculated to be 488, 731, and 642; for the OPC-R1 3562, 2772, 2263 and 3354; for the white prototype device 997. These values are in µg/m^3^ of the PM monitor output and not the true concentrations, which could only be calculated after the final correction due to the uncertainty of the PM monitors. Additionally, the operation range of the PM monitors are given during the correction model verification. For the PM monitors where no negative correlation was observed, the maximal concentrations were above 5000 µg/m^3^ true concentrations.

After the concentration maximum of the PM monitors was defined, the dataset was normalized. This was required to allow for the linear modelling to take place in later steps. Therefore, the best normalization method was calculated for each PM monitor (Table S2). Based on this, a log10-transformation was used to normalize the dataset and allow for fitting linear mixed-effect models.

### 3.3. Evaluation Variable Analysis

Results of the parameter selection are shown in Figure 3 and correction model curves are shown in Figures S2–S8.

Between-device variation showed only relevant effects for the OPC-R1 sensors (1.05-fold improvement of model accuracy). This suggests that for this monitor type, more natural variation in the data output is present between different units. This also indicates that for the other PM monitor types, one evaluation may be suitable for all PM monitors of that type.

For within-device variation (drift), only minor effects were found for the Airveda and black monitors (1.01-fold improved model accuracy). Because these effects were minimal, within-device variation was deemed not relevant for the tested PM monitors.

The effect of each reference material on the monitor readings was evident for all monitors, with especially large effects for the AirBeam2 and black prototype (1.98 and 2.02-fold increases in model accuracy, respectively) monitors. This confirms that PM monitors react differently to different materials, as was expected.

Temperature was found to be relevant for the Awair omni, AirBeam2, black and white prototype monitors (1.00, 1.02, 1.01 and 1.04-fold increased accuracy, respectively). Although more than half of the tested PM monitor types were affected by changing temperature, effects are minor compared to other variables. This suggests that, for these PM monitors, temperature variations are a lesser source for inaccuracy.

Humidity was relevant for the AirBeam2 and OPC-R1 with 1.04 and 1.02-fold increased accuracy, respectively. This also suggests that, in this case, changing humidity is also of lesser importance for monitor evaluation.

The exposure pattern had a relatively large effect on the accuracy of the AirBeam2 and black prototype (1.33 and 1.27-fold increase in accuracy, respectively), a minor effect for the Airveda and OPC-R1 (1.04 and 1.05-fold increase in accuracy, respectively) and no relevant effects for the Awair omni and white prototype. Together this shows that the exposure pattern may have large effects on accuracy, but this effect is specific for some PM monitor types.

Power supply was only relevant for the black prototype monitor and had low effects on monitor accuracy (1.01-fold increase). Therefore, this variable may be unspecific for PM monitors.

### 3.4. Correction Model Verification

After analysis of the individual evaluation variables, a correction model was generated for each monitor type that included all relevant evaluation variables. These models provided the final uncertainty for each monitor at multiple concentration ranges (Table 4). The target accuracy of 50% uncertainty was achieved for the Awair omni, Airveda and OPC-R1 only at concentrations between 0–500 µg/m^3^. The uncertainty of the white prototype was only below 50% at concentration between 500–1000 µg/m^3^. The AirBeam2 and black prototype performed best, with acceptable uncertainties between 0–2000 and 0–1500 µg/m^3^. Additionally, these monitors had the lowest uncertainties over the whole informative range of 0–5000 µg/m^3^, namely 100 and 120%, compared to the other PM monitors, which show very large uncertainties of up to 300%. Monitors with such high level of uncertainties are unsuitable for monitoring occupational exposure under the tested conditions at these concentrations. These results show that the uncertainty of the PM monitors evaluated here generally increase as the PM concentration increases and that some of the PM monitors tested are incapable of accurately measuring PM at occupationally relevant concentrations.

**Figure 4:**
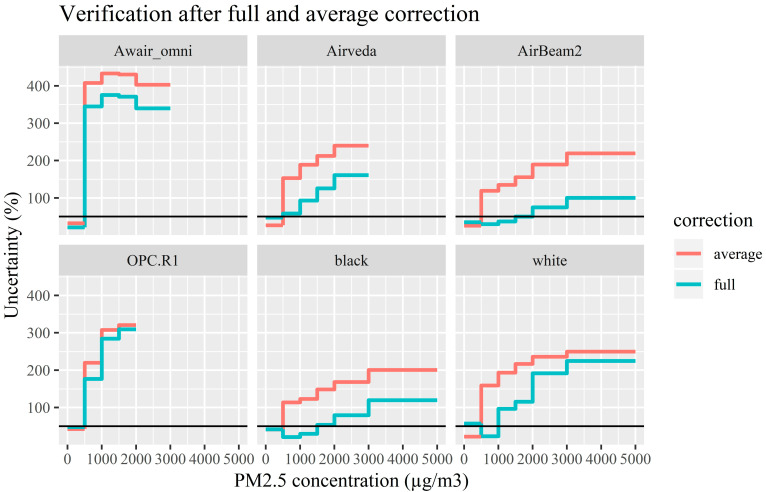
Verifications results after applying the correction model developed in this study (full) and an average correction. Uncertainty measures for each monitor over the concentration bins, where a low uncertainty represents a more accurate monitor. The horizontal black line indicates the target accuracy, set at 50%.

Additionally, the effectiveness of the evaluation variables taken into consideration in the correction model was verified by testing correction models which did not include the evaluation variables (Figure 4). Substantial differences were observed for some of the monitors when the evaluation variables were excluded. To gain more insight in the improvement of accuracy, the ratio of the uncertainty measures of the two correction methods was calculated for each bin. This showed that the accuracy at these concentrations was minimally improved for the OPC-R1 and Awair omni (average of 1.07 and 1.24-fold, respectively), but substantially for the Airveda, white prototype monitor, AirBeam2 and black prototype monitor (average 1.68, 2.26, 2.70 and 2.85-fold, respectively). These results demonstrate the added value of considering relevant variables potentially influencing the performance of the PM monitors and underlining the need for fit-for-purpose evaluation procedure.

## 4. Discussion

The increased use of affordable, non-specialist, user-friendly, time-resolved sensor monitors alternatives to traditional weighted average sampling methods for PM could bring many advantages to the field of occupational exposure controls and monitoring but it may increase the risk of deploying poorly evaluated PM monitors in the workplace. This study exemplifies the benefit of evaluating PM monitors prior to full deployment for occupational exposure monitoring purposes. The methods detailed in this study may be a first step towards a more harmonized strategy for the evaluation of low-cost devices.

Seven evaluation variables were identified and tested for the PM monitors selected. Two variables were found to affect the accuracy of the monitor the most. For all PM monitors, the reference material used for the evaluation was the main contributing variable. Since the reference materials all shared the same particle size distribution, the effect on the readings can only be attributed to the different physical properties of the materials, such as density and refractive index. This shows the need for careful reference material selection. It also shows that the evaluations need to be undertaken for materials comparable to the PM to be monitored in the workplace. The large effect of the exposure pattern indicated that PM monitors may react differently for transient exposure concentrations. This suggests that transient exposure in the workplace should be identified and evaluated prior to deployment. For the other variables, overall only minor effects were found.

None of the monitors tested were sufficiently accurate throughout the whole predefined concentration range of 0–5000 µg/m^3^ respirable dust fraction. However, the AirBeam2 and black prototype monitor performed best after correction, showing uncertainties below the target accuracy of 50% at concentrations up to 2000 and 1500 µg/m^3^, respectively. The Airveda and white prototype monitor showed slightly lower performance, with uncertainties increasing quicker as concentrations increase. The Awair omni and OPC-R1 monitors showed high uncertainties and a low applicable concentration range. In general, the evaluation confirms the expectation that these monitors are not suitable for regulatory exposure assessments or verification of the compliance with occupational exposure limit values, which would require even higher accuracies than the currently set targets. Still, the PM monitors could be used for other specific purposes such as identification of exposure peaks or work activities with relatively higher risk. The monitors could, for instance, be used in a tiered approach where the low-cost devices are used as a general screening tool to detect high-exposure situations. Then, more accurate approaches can be deployed to reliably assess the situation and compare with legal standards. This would result in more efficient monitoring of exposure in the workplace. The evaluation in this case can be used to define in which concentration ranges a PM monitor can operate with sufficient accuracy for screening purposes and above which concentrations more accurate methods have to be used.

It should be noted that these devices are not marketed specifically for occupational use. Further technological or statistical improvements are needed for PM monitors to accurately operate in relevant occupational setting concentration ranges. Possible improvements could include adding an aerosol diluter in the device to reduce the sensor overstimulation. However, this would add another source of error and may increase the lower limit of detection. The inlet of the low-cost device could also be fitted with a particle size selector such as a miniaturized respirable cyclone or a PM4 impactor plate. However, the device would need to be an active rather than a passive sampling device. Another method may be more sophisticated statistical processing of the data, for instance by reporting moving averages instead of raw measurements. However, more investigation is needed for this technique for these purposes.

The required accuracy and operating concentrations can be defined based on the particular aims of the deployment or application. The work undertaken in this study showed that the PM monitor uncertainty generally increased as the PM concentration increased (well above what these monitors were expected to monitor for air quality purposes), suggesting a tradeoff between PM monitor accuracy and reliable measurable concentrations in the workplace. In other words, if the aim of the deployment requires high accuracy monitoring, evaluations may be performed at lower concentrations. Or if relatively high concentrations are to be measured (e.g., workplace settings), a relatively high target accuracy should be set.

The Awair omni and Airveda monitors have internal, manufacturer-set concentration maximums (2830 and 2880 µg/m^3^ true PM_2.5_ concentrations derived from the APS, respectively) above which measurements are not reported. For the Awair omni, this limit was lowered to remove negative correlations. Still, these true values were well above the limits that the manufacturers specified (1000 and 770 µg/m^3^ true PM_2.5_ for the Awair omni and Airveda, respectively). Similarly, the upper concentration limit for the AirBeam2 was 500 µg/m^3^ true PM_2.5_, while the experiments reached far higher concentrations without negative correlations to the reference. Together, this shows that the device effective concentration range may differ between applications and should be confirmed experimentally.

At the time of the study, there were no low-cost PM monitors commercially available that could report occupationally relevant size ranges, such as respirable fractions. Therefore, devices reporting PM_2.5_ were selected for evaluation. The PM_2.5_ size fraction could be used as a relevant indicator for occupational exposure monitoring, or the respirable fraction could be derived from the PM_2.5_ and PM_10_ measurements. Alternatively, the PM monitors could be evaluated against and compared to a real-time reference instrument or sampler that measures, for instance, respirable fraction based concentrations. This would result in a correction model that predicts respirable concentrations based on PM monitor measurements. This was not done for the current study, since this would result in an additional source of variation to the evaluation, while this study was more focused on the intrinsic uncertainty related to the devices.

Comparing the correction models with and without the evaluation variables highlighted the benefit of correcting the evaluation variables as accuracies improved up to 3-fold in relevant concentration ranges. This shows that if the workplace environment may be encapsulated in appropriate evaluation variables, then the effect of these variables on the monitors’ readings can be efficiently corrected for. However, how these variables are identified may be a complex process and differences in this process undertaken by different PM monitor users is likely to result in different outputs. This further underlines the need for a more harmonized device evaluation strategy, so that appropriate monitor selection is ensured before deployment in the workplace, and all factors are properly considered. This study also provided an elaborate example of statistical correction methods for devices evaluated in laboratory conditions. However, one should note that other valid approaches are available, for example based on machine-learning principles [28,29,30,31]. More investigation is required to determine the influence of different statistical methods and which method is most suitable for the evaluation and correction of occupational PM monitors readings.

One limitation of the presented study is that the evaluation is performed in a single laboratory. For a more robust evaluation, experiments should be repeated using multiple experimental setups and the results compared. This could be done, for instance, in a round-robin study. Another possible limitation is that, since sensing technologies are continuously evolving, the currently identified variables will not necessary be relevant for new generations of PM monitors. Still, the currently described methodology could be used to test new variables. Furthermore, no information is available on how this laboratory evaluation compares with an occupational setting evaluation. The accuracy of the tested monitors should also be tested in a workplace for a specific application to determine how well the laboratory evaluation simulates the work environment. Finally, long term drift is a variable that is expected to affect PM monitor accuracy but has not been tested in the current evaluation. Prolonged PM exposure may deteriorate the sensing optics in the devices and reduce accuracy. This could be tested by performing a full evaluation prior to deployment of the monitor and periodic reevaluations while the monitor is being deployed. If the reevaluation is significantly different from the initial evaluation, the PM monitor should be replaced or the correction model should be adjusted.

The evaluation presented in this study has been built upon protocols that have been developed for (PM) monitor use in (occupational) exposure assessment. Spinelle et al. [14] reported an extensive workflow for evaluation of gas monitors for environmental purposes, providing excellent insights in general needs for monitor evaluation. This includes requirements for testing of responsiveness, short and long-term drifts, inferences and intermediate data quality checks. However, it does not cover the specific context that is required for optical PM monitors or uses in occupational settings. Similarly, a technical report by NIOSH [15] gives detailed descriptions on evaluation of direct-reading gas monitors, including statistical approaches, R code and proposed accuracy criteria, but also lacks the specificity for PM monitors. The CEN norm CEN/TR 16013 describes a guide for the use of direct-reading instruments for aerosol monitoring in occupational settings. The first part of this norm [16] describes possible uses for PM monitors and criteria for each use. The second [17] and third [18] parts give technical details on monitor use and statistical approaches for data analysis. While device evaluation is mentioned, no description is given on how to perform the experimental assessment. Finally, ISO norm 11095 [19] describes a basic method for linear calibration correction using reference materials, including concrete requirements such as experimental replicate numbers, but lacks the specificity for optical (PM) monitors and occupational environments. Together, this shows that some information for occupational evaluation of PM monitors is available, but it is decentralized and needs to be translated for this specific purpose. The evaluation in the current study combines the relevant aspects of related protocols with novel principles based on expert judgement (e.g., multiple exposure patterns) to generate an evaluation that is both specific for PM monitors and specific for the highly dynamic occupational exposure assessment.

Altogether, the results in this study show that PM monitor evaluation is influenced by multiple factors, which are closely related to the characteristics of the PM generated in the workplace of interest. These initial findings could be integrated into broader guidelines on how to harmonize the laboratory evaluation of PM monitors for occupational exposure monitoring. Such guidelines should consider the different possible applications of PM monitors and provide users with insights in tailoring the evaluation strategy to fit their specific workplace application. In order to develop such a document, the robustness of the current strategy should be tested. We are suggesting that this could be done by repeating this evaluation within different laboratories and broadening the analysis of the current dataset using different methods.

## 5. Conclusions

Six low-cost PM monitors to be used for occupational exposure monitoring were evaluated in laboratory conditions. Seven evaluation variables likely to affect the readings were identified through literature and expert opinions. Testing of the variables revealed that the reference material and exposure pattern were the main factors influencing PM monitor performance, with other variables having minor effects. Accuracy of the PM monitors increased up to 3-fold upon correction for these evaluation variables. Not surprisingly, this shows that laboratory evaluation of PM monitors for occupational exposure monitoring should be tailored to the intended use and application scenario. To ensure this device evaluation is performed in a harmonized way and agreed upon method, we are suggesting that a generic evaluation guideline should be developed, which could build upon the currently presented evaluation strategy.

## Figures and Tables

**Figure 3:**
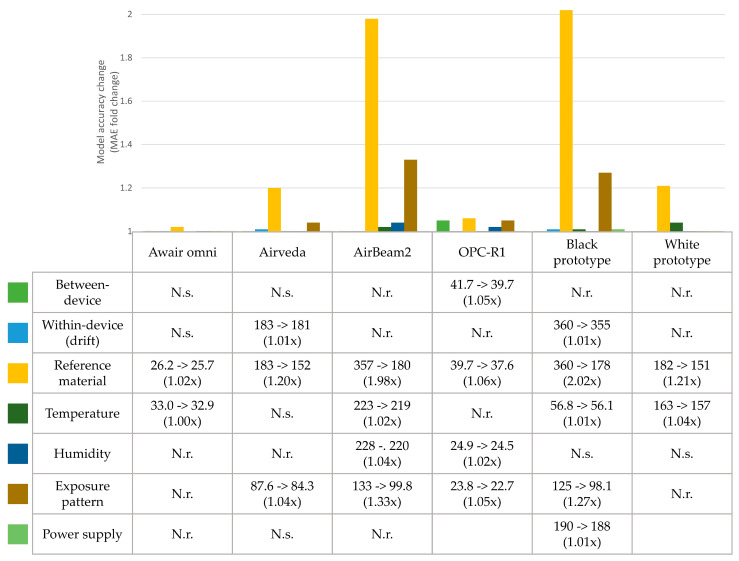
Parameter selection results. Values represent the absolute (in µg/m^3^) and fold changes in mean absolute error (MAE) in upon inclusion of the variable in the model. N.s. represents not significant variables (*p* > 0.05) and N.r. represents not relevant variables (e.g., the MAE was not decreased).

**Table 1:** Experimental design ^1^.

Name Parameter	Value(s)
Concentration range	0–5000 µg/m^3^ respirable PM
Target accuracy	≤50% uncertainty
Target responsiveness	1 min

^1^ Experimental design parameters relate to the experimental procedures and targets for the evaluation.

**Table 2:** Evaluation variables.

Name Parameter	Value(s)
Reference material	Arizona road dust A2, SiO_2_ A2 and Al_2_O_3_ A2
Within-device variation	Repeat measurements at t = 0, ~24 and ~48 h
Between-device variation	≥3 replicate units
Power supply	Battery and wired
Temperature	15, 20 and 25 °C
Relative humidity	25, 50 and 75% RH
Exposure pattern	Transient and stable

**Table 3:** Technical specifications of monitors evaluated.

Monitor	Manufacturer	Size (l × b × h, cm)	PM Sensors (PM Sizes)	Concentration Range (µg/m^3^)	Other Sensors	Measurement Frequency (s)	Other Features
AirBeam2	Habitatmap, Brooklyn, NY, USA	10 × 3 × 15	Plantower PMS7003 (PM_1_, PM_2.5_, PM_10_)	0–500	Temperature (MCP9700T-E/TT), humidity (Honeywell HIH-5030-001)	1	Open source, wearable
Airveda	Airveda, Ghaziabad, India	14 × 5 × 14	Unknown (PM_2.5_, PM_10_)	0–1000	Temperature, humidity, CO_2_	30	Static monitor, not wearable
Awair omni	Awair, San Francisco, CA, USA	10 × 4 × 10	Unknown (PM_2.5_)	0–1000	Temperature, humidity, CO_2,_ VOCs, light, noise	~10	Static monitor, not wearable
“White” prototype	Isensit (Eindhoven, the Netherlands)	7 × 3 × 7	Plantower PMSA003 (PM_1_, PM_2.5_, PM_10_)	0–1000	Temperature, humidity, light, noise	10	Wired powered only
“Black” prototype	Isensit	5 × 3 × 7	Plantower PMS5003 (PM_1_, PM_2.5_, PM_10_)	0–1000	Temperature, humidity	10	Wearable
Sensor only	Alphasense, New York, NY, USA	7 × 5 × 7	OPC-R1 (PM_1_, PM_2.5_, PM_10_)	Up to 10.000 particles/s	Temperature, humidity	1	Sensor only, wired powered only.

**Table 4:** Verification results expressed as relative uncertainty (%).

Concentration Range (µg/m^3^)	Awair Omni	Airveda	AirBeam2	OPC-R1	Black	White
500	21.1	47.2	35	47.5	41.7	57.2
1000	345	58.3	29.7	177	21	22.9
1500	376	93	37	284	29.7	96.6
2000	371	126	50	309	53.8	115
3000	340	161	74.7	NA	79.3	192
5000	NA	NA	100	NA	120	225

Verification is quantified as the relative uncertainty (in %) of a particulate matter (PM) monitor within a specific concentration bin. The left column indicates the upper limit of the PM_2.5_ concentration range bin in absolute values according to the APS reference. The remaining values are the uncertainty measures for each PM monitor. NA: Not available due to concentration limits of PM monitors.

## Supplementary Materials

The following are available online at https://www.mdpi.com/1660-4601/17/22/8602/s1, Table S1: List of experiments, Table S2: Linearization of the evaluation dataset, Figure S1: Experimental setup, Figure S2: Between-device variation analysis, Figure S3: Within-device variation (drift) analysis, Figure S4: Material variable analysis, Figure S5: Pattern variable analysis, Figure S6: Power variable analysis, Figure S7: Temperature variable analysis, Figure S8: Humidity variable analysis.
