# Supplementary material for: Exploring Evaluation Variables for Low-Cost Particulate Matter Monitors to Assess Occupational Exposure
Source: Int J Environ Res Public Health. 2020 Nov 19;17(22):8602. doi: 10.3390/ijerph17228602 (PMC7699371; doi:10.3390/ijerph17228602)
Supplement: Supplementary file 1 [file ijerph-17-08602-s001.pdf]

Supplementary information

Exploring evaluation variables for low-cost particulate matter monitors to assess occupational exposure

Sander Ruiter [1,*], Eelco Kuijpers [1], John Saunders [2], John Snawder [3], Nick Warren [2], Jean-Philippe Gorce [2], Marcus Blom [1], Tanja Krone [1], Delphine Bard [2], Anjoeka Pronk [1] and Emanuele Cauda [3]

[1] Netherlands Organization for Applied Scientific Research (TNO), 3584 CB Utrecht, The Netherlands; sander.ruiter@tno.nl
[2] Health and Safety Laboratory (HSL), SK17 9JN Harpur Hill Buxton, UK; John.Saunders@hse.gov.uk; Nick.Warren@hse.gov.uk; Jean-Philippe.Gorce@hse.gov.uk; Delphine.Bard@hse.gov.uk
[3] Centers for Disease Control and Prevention, National Institute for Occupational Safety and Health (NIOSH); jts5@cdc.gov; cuu5@cdc.gov
* Correspondence: sander.ruiter@tno.nl

Received: date; Accepted: date; Published: date

Supplementary tables

Table S1. List of experiments

Material	Exposure pattern	Power supply	Short-term drift (h)	Temperature (°C)	Relative humidity (%)	Comments
ARD	stable (~333 µg/m3)			20	50	Gravimetric sampling
ARD	stable (~666 µg/m3)			20	50	Gravimetric sampling
ARD	stable (~1000 µg/m3)			20	50	Gravimetric sampling
ARD	stable (~2500 µg/m3)			20	50	
ARD	stable (~5000 µg/m3)			20	50	
ARD	transient	wired		20	50	
ARD	transient	battery	0	20	50	
ARD	transient		16	20	50	
ARD	transient		40	20	50	Gravimetric sampling
ARD	transient			15	50	
ARD	transient			15	25	Gravimetric sampling
ARD	transient			15	75	
ARD	transient			20	25	
ARD	transient			20	75	
ARD	transient			25	50	
ARD	transient			25	25	Gravimetric sampling
ARD	transient			25	75	
Al_2O_3	stable (~666 µg/m3)			20	50	Gravimetric sampling
Al_2O_3	stable (~1000 µg/m3)			20	50	Gravimetric sampling

Al_2O_3	stable (~2500 µg/m3)			20	50	Gravimetric sampling
Al_2O_3	stable (~5000 µg/m3)			20	50	Gravimetric sampling
Al_2O_3	transient	wired		20	50	
Al_2O_3	transient	battery	0	20	50	Gravimetric sampling
Al_2O_3	transient		19	20	50	
Al_2O_3	transient		41	20	50	
Al_2O_3	transient			20	75	
Al_2O_3	transient			15	50	Gravimetric sampling
Al_2O_3	transient			15	75	
Al_2O_3	transient			25	25	
Al_2O_3	transient			25	50	Gravimetric sampling
Al_2O_3	transient			25	75	
SiO_2	stable (~ 2500µg/m3)			20	50	Gravimetric sampling
SiO_2	stable (~ 5000µg/m3)			20	50	Gravimetric sampling
SiO_2	transient	wired		20	50	
SiO_2	transient	battery	0	20	50	
SiO_2	transient		16	20	50	
SiO_2	transient		47	20	50	
SiO_2	transient			20	75	Gravimetric sampling
SiO_2	transient			15	50	
SiO_2	transient			15	75	Gravimetric sampling
SiO_2	transient			25	25	
SiO_2	transient			25	50	Gravimetric sampling
SiO_2	transient			25	75	

List of experiments that were performed during the evaluation. Blank spaces represent that the variable was not of interest in that experiment. In this case, the variable was at average settings: battery powered, 20 °C and 50% RH. If one gravimetric sample was collected for multiple experiments, this is shown by a vertical line. ARD: Arizona road dust.

Table S2. Linearization of the evaluation dataset.

Monitors	No transform	$\log 10\ (x)$	Box Cox	Yeo-Johnson	$arcsinh(x)$	$\sqrt{x}$
Awair_omni	37.9	3.5	3.2	9.4	9.1	16.2
Airveda	14.8	2.0	1.9	4.2	4.0	6.3
AirBeam2	314.2	19.1	21.7	65.0	67.4	104.3
OPC-R1	214.0	9.7	8.7	67.3	91.7	95.5
black	52.0	7.0	7.6	15.5	15.9	32.8
white	16.5	3.4	2.7	5.8	5.5	7.9

Values in the table represent results from the bestNormalize function, given as Pearson P statistics divided by its degrees of freedom. Smaller values indicate more normal distributions.

Supplementary figures

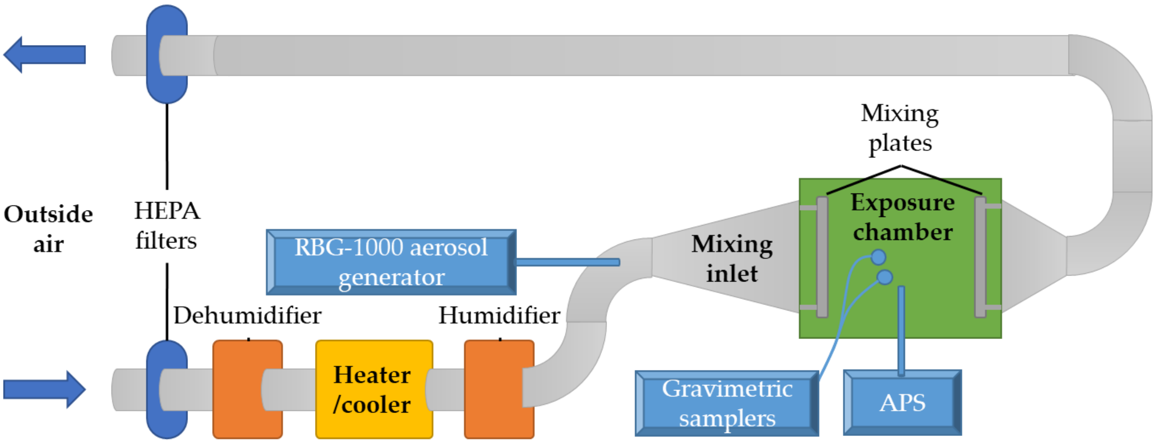

Figure S1. Experimental setup. Outside air is taken and filtered to remove all PM and dehumidified to removes water from the air. Next, the air is heated/cooled to the set temperature and humidified to the set relative humidity. Aerosols are added in-stream by an RBG-1000 aerosol generator and dispersed in a broad mixing inlet. Mixing plates near the chamber in- and outlet ensure a homogeneous concentration in the exposure chamber (approximately 120x80x80 cm). Particle concentration measurements are made using an APS placed directly under the chamber, as well as two gravimetric samplers. Air that is passed through the exposure chamber is filtered and released to the outside environment.

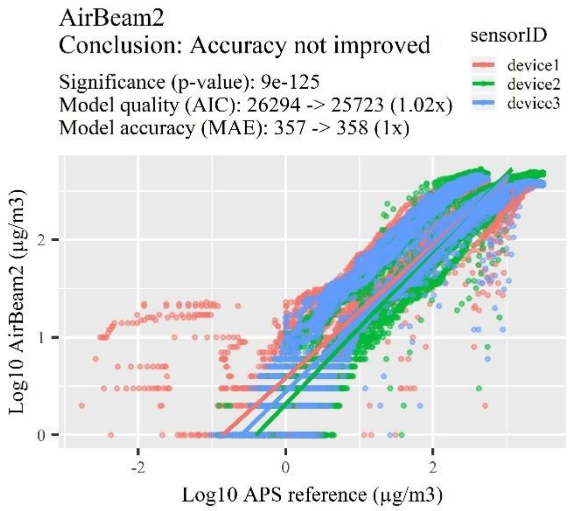

(**a**)

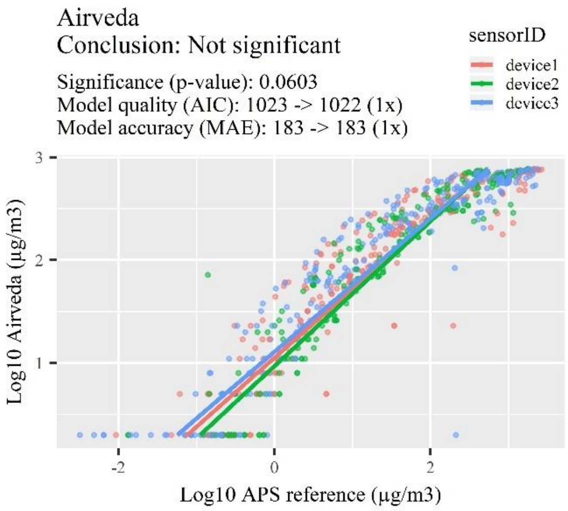

(**b**)

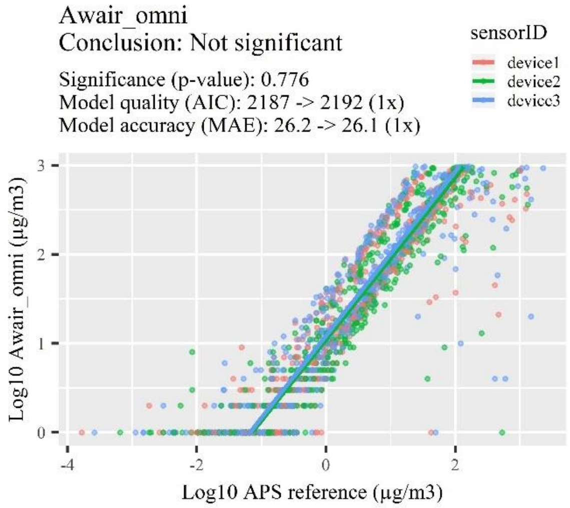

(**c**)

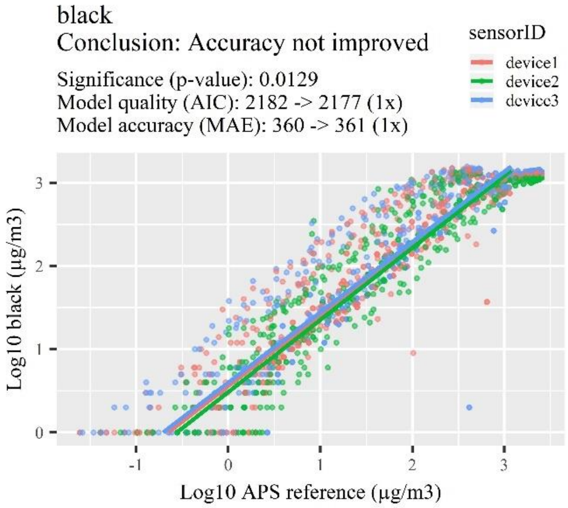

(**d**)

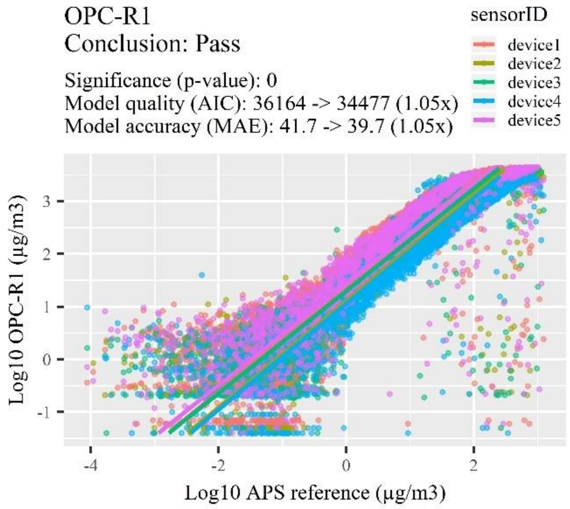

(**e**)

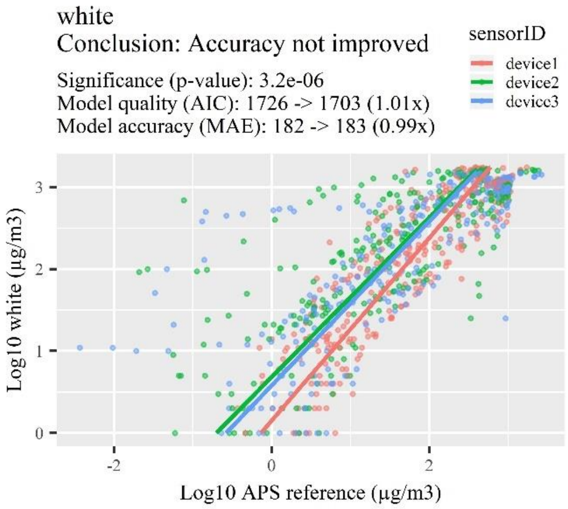

(**f**)

Figure S2. Between-device variation analysis. All experiments were carried out using all three materials, with transient exposure patterns, on battery power at 20°C and 50% relative humidity. Variable was analyzed as a random effect using linear mixed-effects models and compared to a baseline model containing only monitor and reference variables.

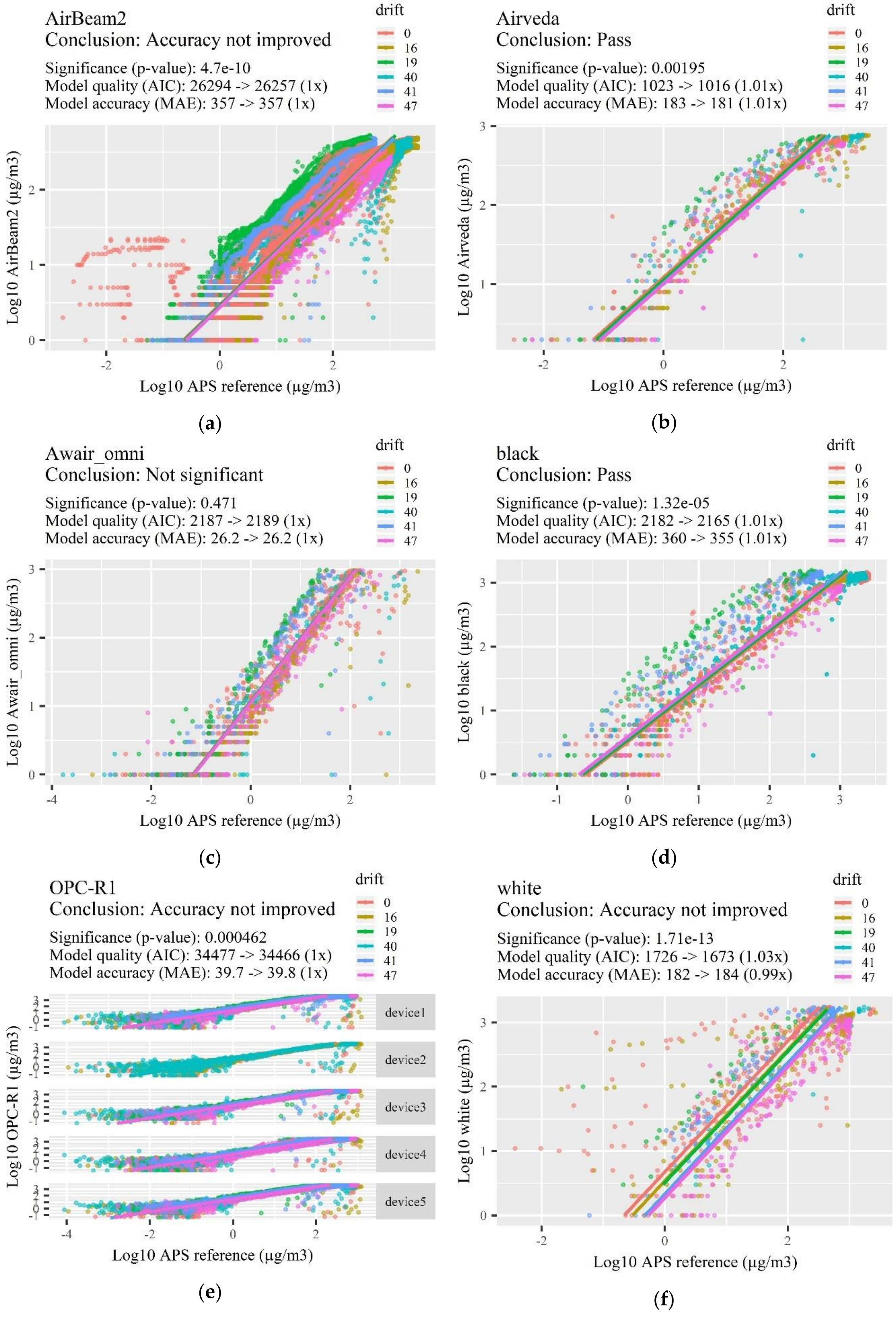

Figure S3. Within-device variation (drift) analysis. All experiments were carried out using all three materials, for all three devices, with transient exposure patterns, on battery power at 20°C and 50% relative humidity. Drift was analyzed as an additional fixed effect using simple linear regression and compared to a baseline model containing only monitor and reference variables.

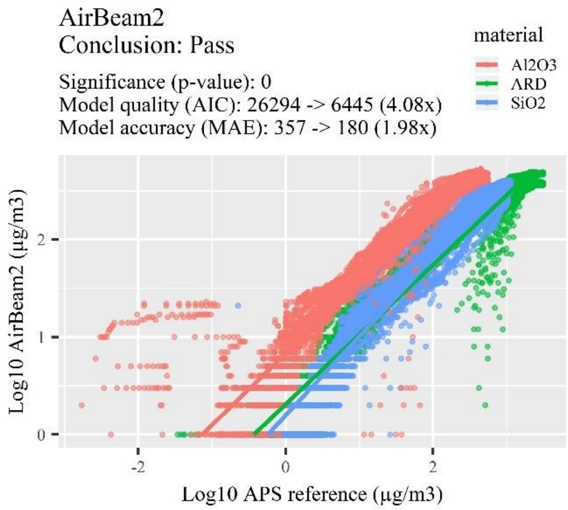

(**a**)

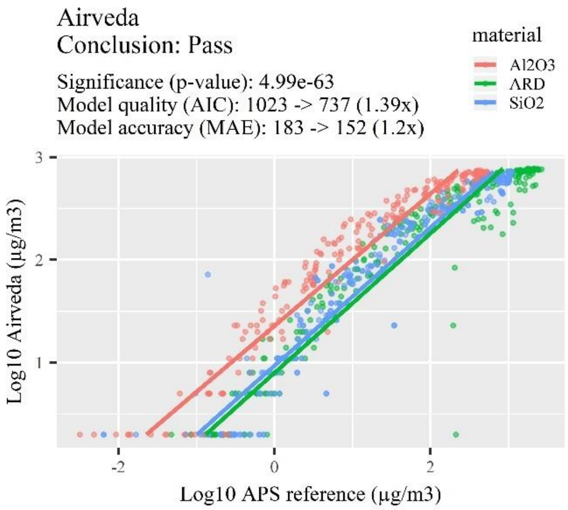

(**b**)

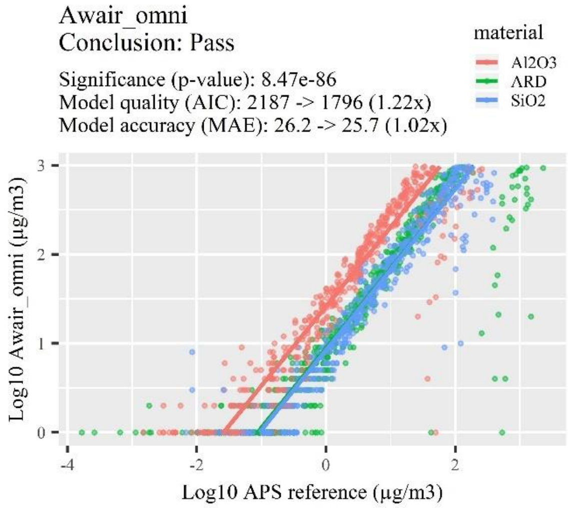

(**c**)

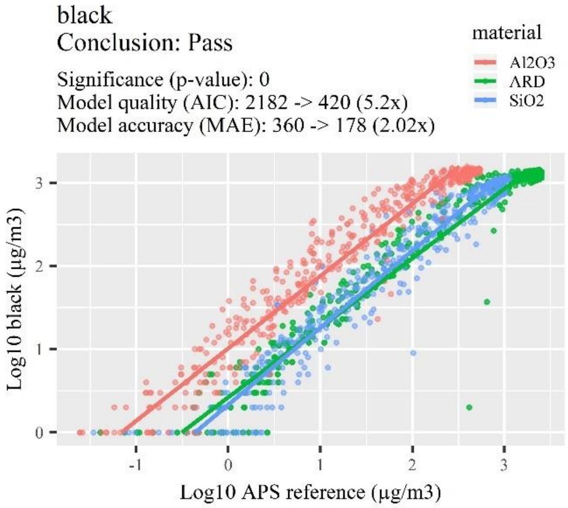

(**d**)

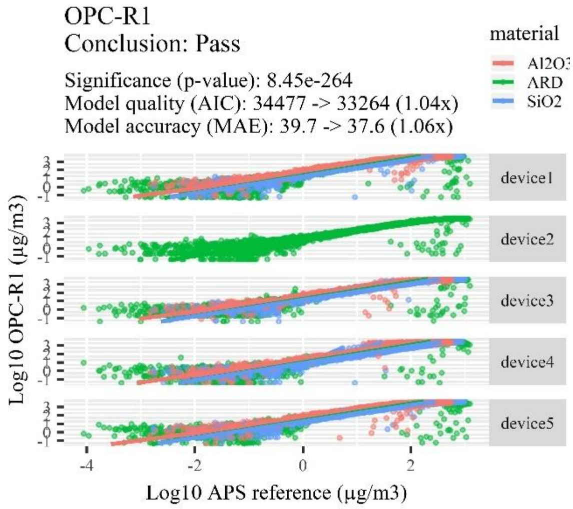

(**e**)

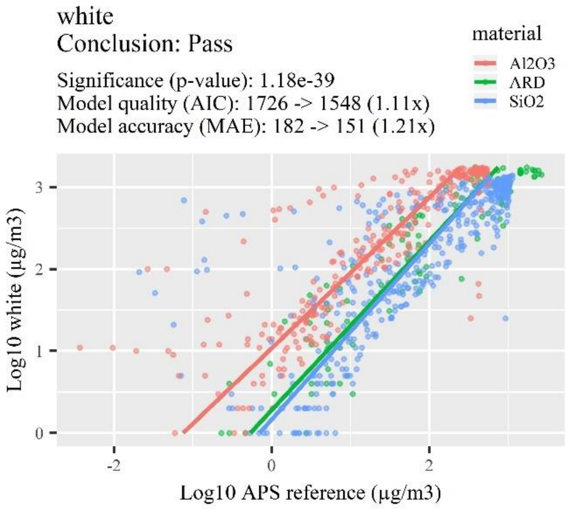

(**f**)

Figure S4. Material variable analysis. All experiments were carried out for two devices (three in case of OPC-R1), with transient exposure patterns, on battery power at 20°C and 50% relative humidity. Variable was analyzed as a random effect using linear mixed-effects models and compared to a baseline model containing only monitor and reference variables.

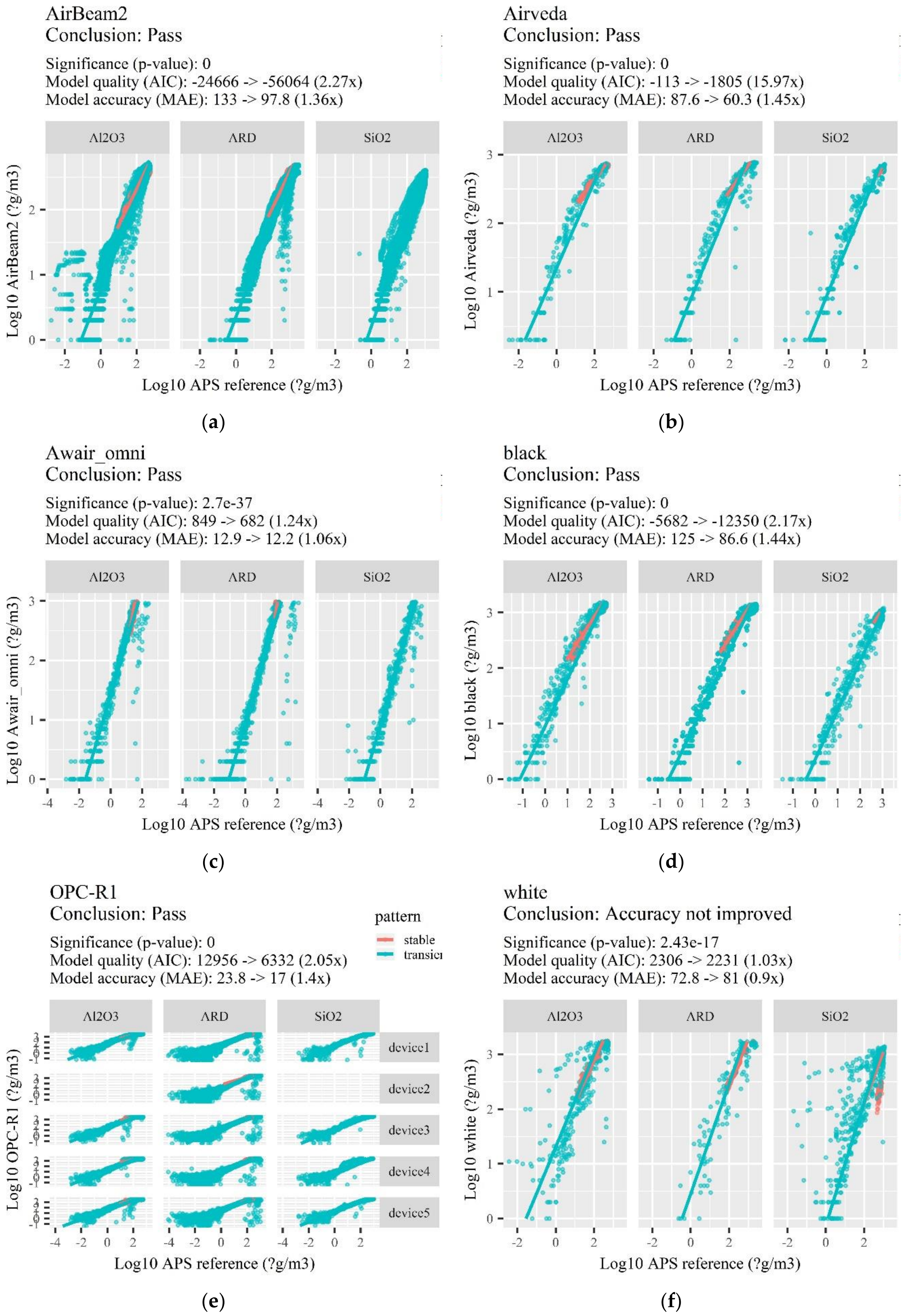

Figure S5. Pattern variable analysis. All experiments were carried out for three materials, two devices (three in case of OPC-R1), on battery power at 20°C and 50% relative humidity. Variable was analyzed as a random effect using linear mixed-effects models (also containing material as a random variable) and compared to a baseline model containing only monitor, reference and material variables.

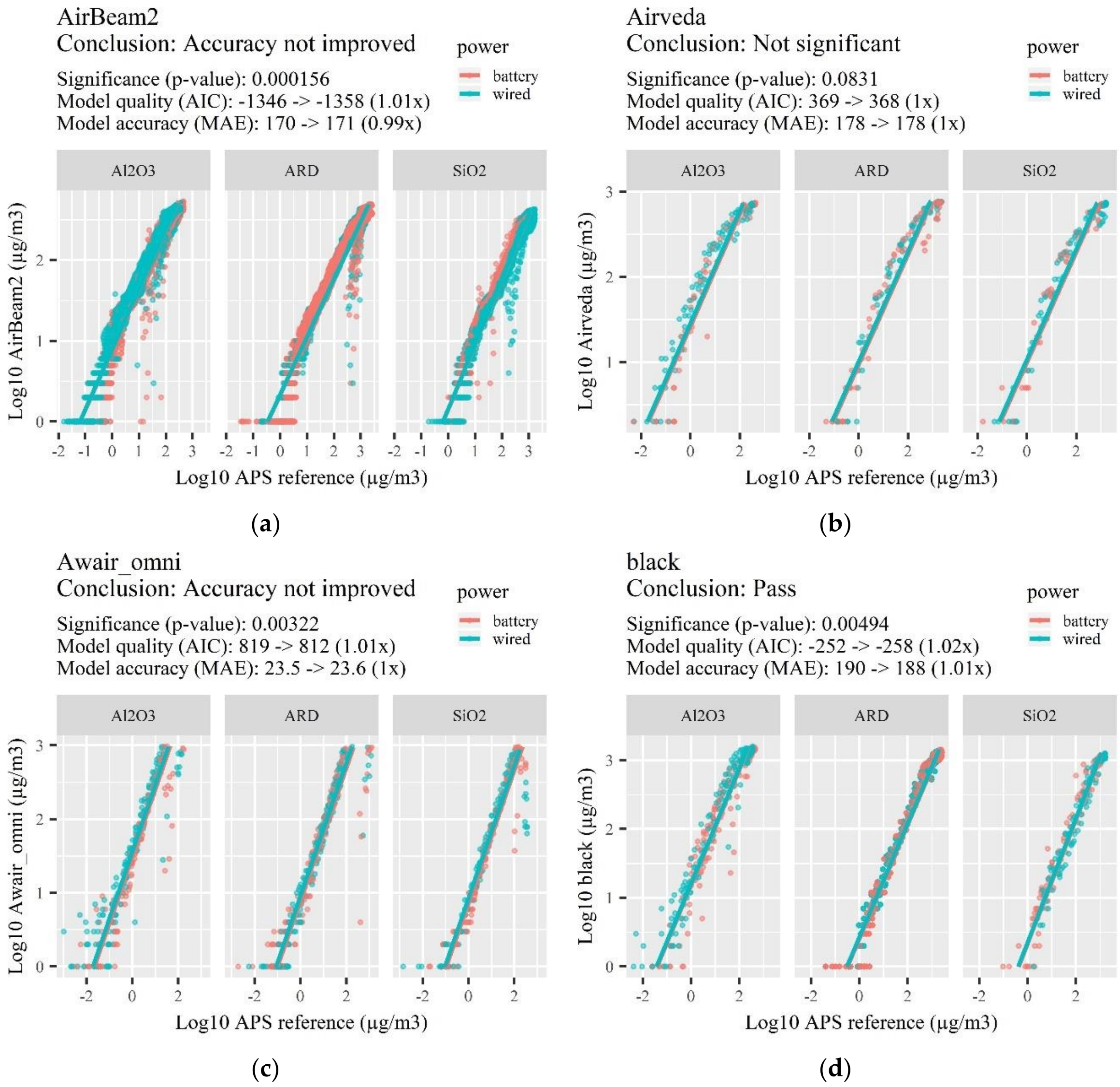

Figure S6. Power variable analysis. All experiments were carried out for three materials, two devices (three in case of OPC-R1), with transient exposure patterns, at 20°C and 50% relative humidity. Variable was analyzed as a fixed effect using linear mixed-effects models (also containing material as a random variable) and compared to a baseline model containing only monitor, reference and material variables. The OPC-R1 and iSensit 'white' did not contain batteries so the variable could not be tested.

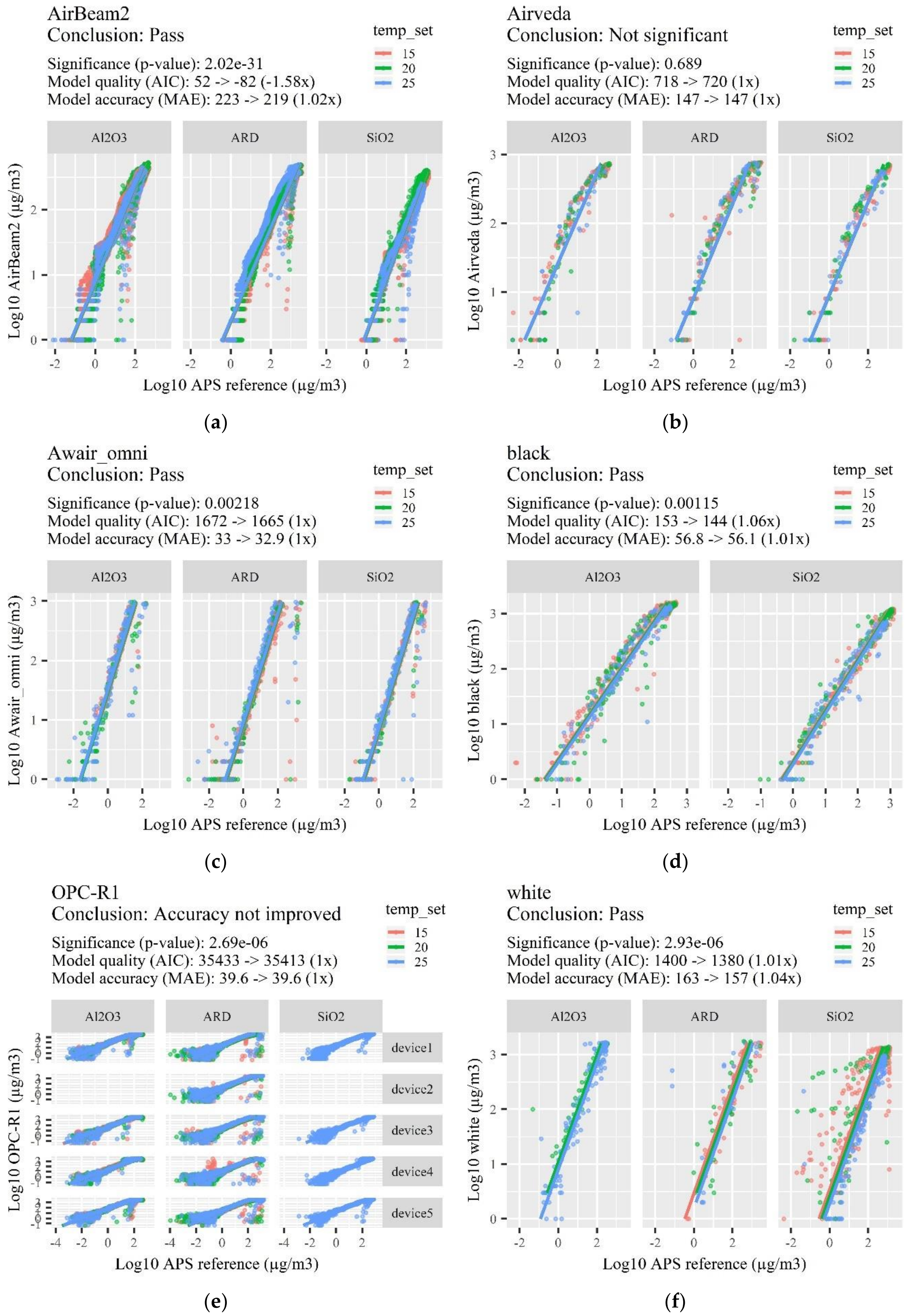

Figure S7. Temperature variable analysis. All experiments were carried out for three materials, two devices (three in case of OPC-R1), with transient exposure patterns, on battery power at 50% relative humidity. Variable was analyzed as a fixed effect using linear mixed-effects models (also containing material as a random variable) and compared to a baseline model containing only monitor, reference and material variables.

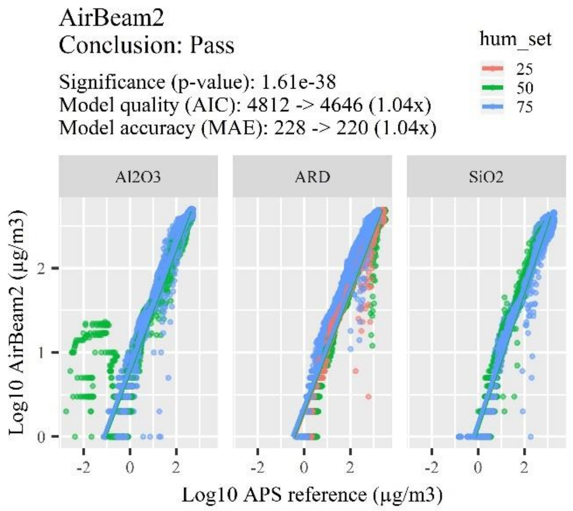

(**a**)

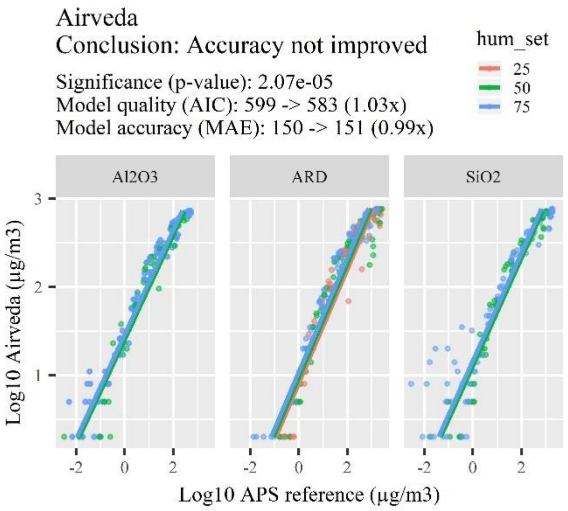

(**b**)

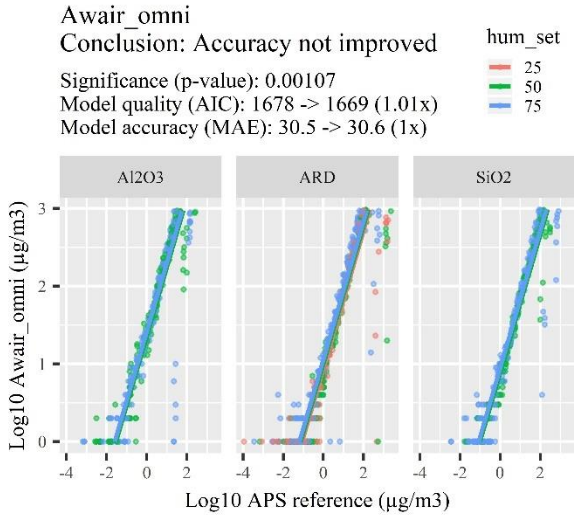

(**c**)

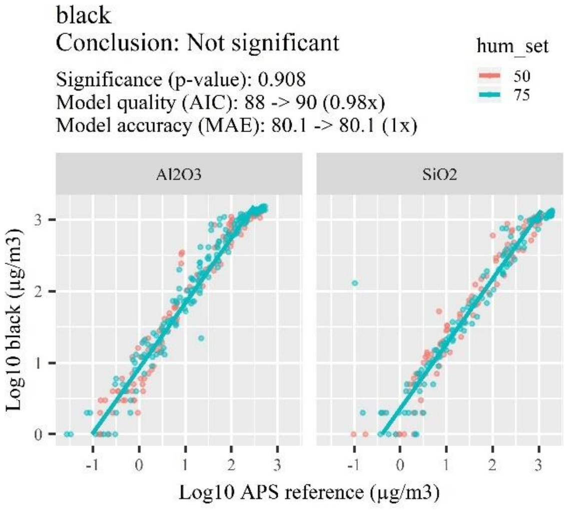

(**d**)

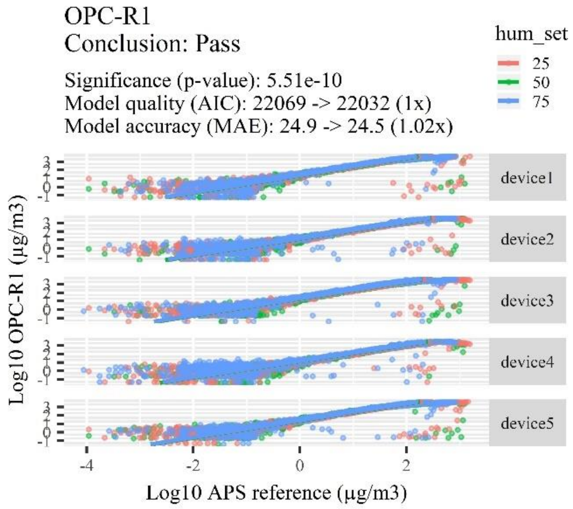

(**e**)

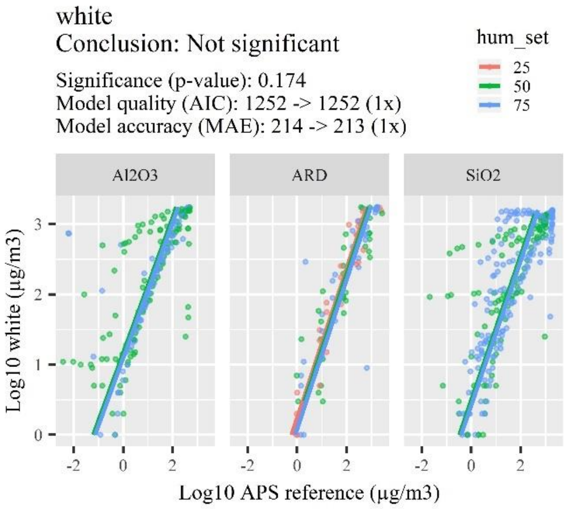

(**f**)

Figure S8. Humidity variable analysis. All experiments were carried out for three materials, two devices (three in case of OPC-R1) , with transient exposure patterns, on battery power at 20°C. Variable was analyzed as a fixed effect using linear mixed-effects models (also containing material as a random variable) and compared to a baseline model containing only monitor, reference and material variables.
